# Production of Bulk Chemicals with Biocatalysis: Drivers and Challenges Reflected in Recent Industrial Granted Patents (2015–2020)

**DOI:** 10.3390/molecules26030736

**Published:** 2021-01-31

**Authors:** Nadia Guajardo, Pablo Domínguez de María

**Affiliations:** 1 Programa Institucional de Fomento a la Investigación, Desarrollo e Innovación (PIDi), Universidad Tecnológica Metropolitana, Ignacio Valdivieso 2409, Santiago de Chile 8940000, Chile; 2 Sustainable Momentum SL., Avenida Ansite 3, 4-6, 35011 Las Palmas de Gran Canaria, Spain; dominguez@sustainable-momentum.net

**Keywords:** biocatalysis, bulk compounds, sustainable processes, granted patents

## Abstract

The application of biocatalysis and White Biotechnology tools in chemical areas concerning the production of bulk compounds and other related low-added value products (with high volumes) has been gaining importance in recent years. The expected drivers of biocatalysis for these sectors are energy savings, regioselectivity (leading to cleaner products), the possibility of using thermolabile substrates, as well as the generation of less by-products and manageable wastes. This paper explores some recent industrial granted patents related to biocatalysis and bulk chemicals. Several patents have been identified in fields such as biodiesel and esterification reactions, and sugar or furan chemistry. Overall, innovative strategies involve the identification of novel enzymes, the set-up of improved immobilization methods, as well as novel reactor designs that can offer improved performances and economics. The reported examples indicate that biocatalysis can certainly offer opportunities for these areas as well, far from the typical pharmaceutical and fine chemical applications often reported in the literature.

## 1. Motivation for Biocatalysis Applied to Bulk Chemicals

White Biotechnology strategies use cells or enzymes as catalysts to transform (un)natural substrates into a broad range of marketable chemicals [1,2]. In recent decades, industrial processes using biocatalysts have boosted, with major applications devoted to the development of (highly valuable) pharmaceutical products [3]. This has been motivated by the advantageous properties of enzymes, such as enantio- [4,5,6], regio- [7,8] and chemo-selectivity [8], together with the fact that the high market value of the products makes the application of the biocatalysts profitable [9]. 

**Figure 1:**
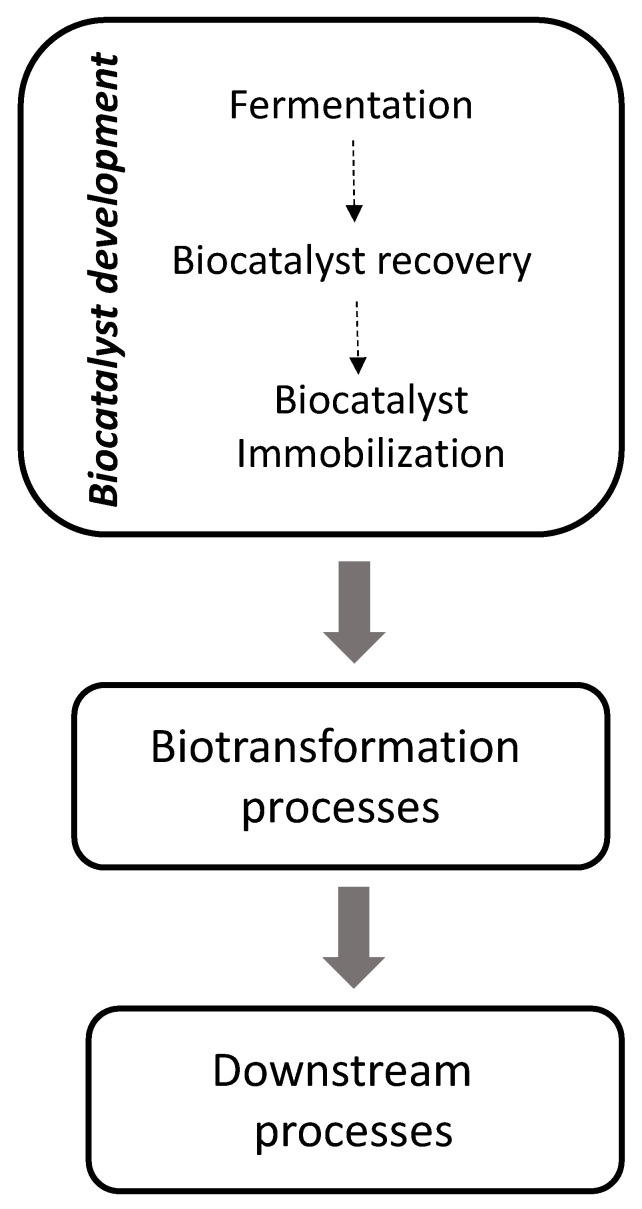
Flow chart for the production of bulk compounds through biocatalysis, depicting the units needed to produce biocatalysts, and subsequent aspects related to the bioprocess.

Aside from pharmaceutical and fine chemical applications, other sectors related to the production of bulk chemicals and less valuable products—with larger volumes—might also benefit from White Biotechnology approaches. In these segments, biocatalysis may bring several drivers to be considered, namely, environmental advantages, energy savings, regioselectivity, possibility of using thermolabile substrates, as well as potential penetration in novel markets (e.g., with natural labelling). Despite that untapped potential, however, applications of enzymes in these areas have traditionally attracted less interest, with only few examples at the industrial level [2]. Primarily, this may be related to the inherent costs associated with the production of biocatalysts, due to the need of numerous unit operations to produce them (Figure 1) [9,10]. A first upstream step—biocatalysis production—is subsequently followed by (bio)process optimization and downstream from typically aqueous solutions, to render the biocatalysts. This may become a challenge which often results in unacceptable overall costs; albeit in some cases, economy of scale may play an advantageous role [11]. Finally, biocatalysts must be immobilized to be (re)used several times to cope with economics of low added-value products in bulk chemical production.

With respect to the use of biocatalysts, the market is steadily increasing. For example, in 2020, it amounted for USD 10.6 billion, and it is expected to reach USD 14.9 billion by 2027 [12]. However, it must be considered that many companies that develop products through biocatalytic processes produce their own biocatalysts, and therefore, these values may vary significantly [13]. Another important aspect to note is that many enzymes are used as additives, for example, in the food and detergent industry, and not as process catalysts [14,15]. The tools of recombinant DNA together with the development of high cell density and fast growth rate are fundamental aspects to be optimized to expand the applications of biocatalysts in industrial scale [16], and especially when less valuable, high-volume molecules are intended. Moreover, to cope with tight economics related to bulk chemical production, enzyme immobilization strategies are often a crucial and powerful tool to modulate the catalytic properties of the enzyme and allow the reuse of the biocatalyst. Many immobilization techniques using different materials as carriers have been reported [17,18,19]. Furthermore, the reuse of the biocatalyst through immobilization allows the operation of continuous reactors, with which bioprocesses with industrial potential could be intensified [20,21,22].

Apart from biocatalyst production and eventual immobilization (typically mandatory when low valuable products are intended), downstream processing must be taken into account as well, considering aspects related to costs, waste production and material use (e.g., which solvent to be used, type of membranes and techniques) [11,23]. As an example, for the development of bulk chemicals, the commonly found unpaired polarities of the reagents may require the use of large volumes of hazardous organic solvents. Herein, more sustainable solvents such as deep eutectic solvents (DES) [24]; biogenic solvents, e.g., Cyrene^TM^ [25]; and in some cases, ionic liquids (IL) (provided that the ecology and the economy of the process is aligned) [26] have great potential to circumvent these issues, provided that economics of the solvents can reach the requested figures.

The penetration of enzyme catalysis in bulk chemicals has witnessed a renaissance in recent years, and biotechnological applications related to biosynthesis, commodity production, biofuels and biorefineries are increasingly appearing. Given that interest, and aiming at establishing some current trends, this article maps out the patent landscape of recent years (2015–2020), with a focus on industrial patents that have been actually granted (to reinforce that some innovative steps are secured). Innovative actions in significant areas, such as biofuels (and biorefineries), sugar chemistry, esterifications and furan valorization, have been identified and will be discussed in the following sections. 

## 2. Recent Granted Patents Applying Biocatalysis for Biofuel Production

In the field of biofuels, innovations related to biocatalysis have mainly focused on the production of biodiesel through (trans)esterifications. The granted patents cover China, United States, South Korea and Taiwan. The strategies in the quest for novelty rely on the identification of new lipases (new sequences) able to conduct (trans)esterifications for biodiesel synthesis under industrially sound conditions. Moreover, a significant number of granted patents focus on the development of new immobilization systems (e.g., carrier-free ones) that may enable the effective reuse of the biocatalyst, as well as on the designing of new processes and process equipment with improved performances for synthesis, downstream processing, etc. 

**Figure 2:**
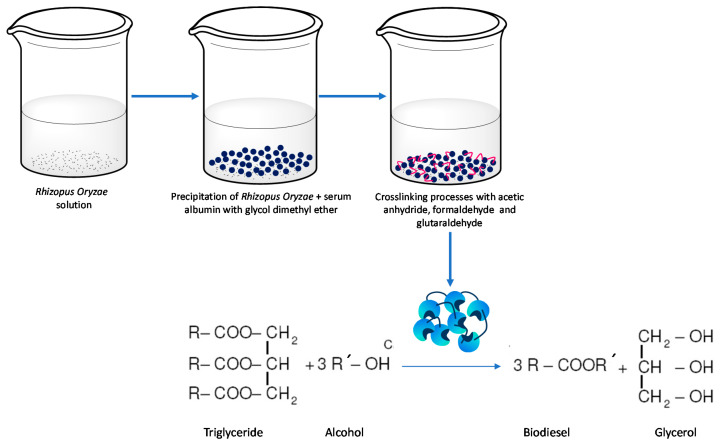
Development of an immobilized biocatalyst for enzymatic transesterification to obtain biodiesel [27].

Regarding immobilization, the use of the strategy of cross-linked aggregates of *Rhizopus oryzae* lipase (Figure 2) led to an increase in the stability of the biocatalyst, due to the use of bovine albumin as an additive with the purpose of increasing the surface lysine residues of the enzyme, for the subsequent interbreeding. Moreover, the presence of glycol dimethyl ether allowed the precipitation of the auxiliary protein and *Rhizopus oryzae* lipase in 20–50 min at 4 °C. The approach resulted in an enhancement of the stability of the derivative. This is important in the production of biodiesel, due to the well-known inactivation of the enzyme by the surplus of methanol. In these patents, the use of vegetable substrates, as well as animal and microbial fats were recommended to be reacted with methanol and using *tert*-butyl alcohol as (inert) reaction media [27].

**Figure 3:**
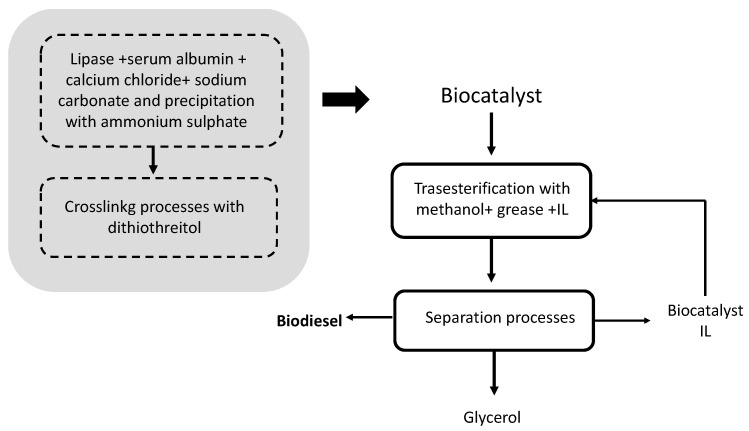
Flow diagram of the process to obtain biodiesel catalyzed by a carrier-free immobilized lipase using ionic liquids (IL) as reaction media [28].

Another immobilization system consists of the formation of calcium carbonate lipase microspheres to subsequently cross-link the molecules with dithiothreitol (DTT), obtaining, after washing and centrifuging, a porous microsphere of immobilized lipases (Figure 3). In the transesterification process per se, some ionic liquids (IL) to assist in the biodiesel separation process were incorporated and blended with grease (Figure 3) [28]. The IL considered were all imidazolia-containing solvents, such as [Bmim][PF_6_], [Bmim][NTf_2_], [Bmim][Tf_2_N], [Emim][Tf_2_N] and [Emim][TfO]. The mass ratio of IL: grease was set at 1:10, while the biocatalyst: grease was 1:10 and the methanol: grease was 3:1. To improve the economy of the entire process, the biocatalyst and the ionic liquid were successfully reused several times [28].

Another technological development is the formation of emulsions to immobilize lipases. The emulsion consisted of a solution of dimethyl silicone polymer, in which *Candida antarctica* lipase B and a platinum catalyst were incorporated to improve the mechanical properties. Subsequently, magnetic vacuum was used to form a gel microsphere which was filtered to obtain the biocatalyst. The substrates used to obtain biodiesel, catalyzed by the designed biocatalyst, were different vegetable oils and methanol or ethanol as alcohols. The innovation claimed by this invention was the formation of a static emulsion, which enabled a homogeneous distribution and high enzyme loadings in the derivative, resulting in a significant increase in the catalytic activity. Moreover, the second advantage of this immobilization methodology is that the system allows a better performance of the biocatalyst at the oil–water interface, together with a straightforward reuse [29].

Furthermore, another recently granted patent discloses the immobilization of the fungus *Rhizopus oryzae* on luffa fibers using glutaraldehyde as a crosslinking agent to covalently bind the microorganisms to the fibers. The potential advantages of this technology are the use of biobased raw materials and their straightforward preparation. The objective of using immobilized cells instead of immobilized lipases is that the immobilized cells resulted catalytically more stable than the isolated lipases, being able to be reused several times in the transesterification reaction to obtain biodiesel [30]. 

Apart from the immobilization systems that may lead to improved enzymatic derivatives for biodiesel synthesis, the identification of novel enzymes has also been reported in the area. For example, a specific monoacylglycerol lipase from *Arabidopsis thaliana* plant, cloned and expressed in *E. coli*, has found applications for the synthesis of biofuels and other biomaterials [31]. In a different area, another example is the production of biodiesel by using a recombinant lipase from *Candida rugosa* expressed in *P. pastoris*, which allows the development of high-density cultures, reducing the production costs of the lipase [32].

**Figure 4:**
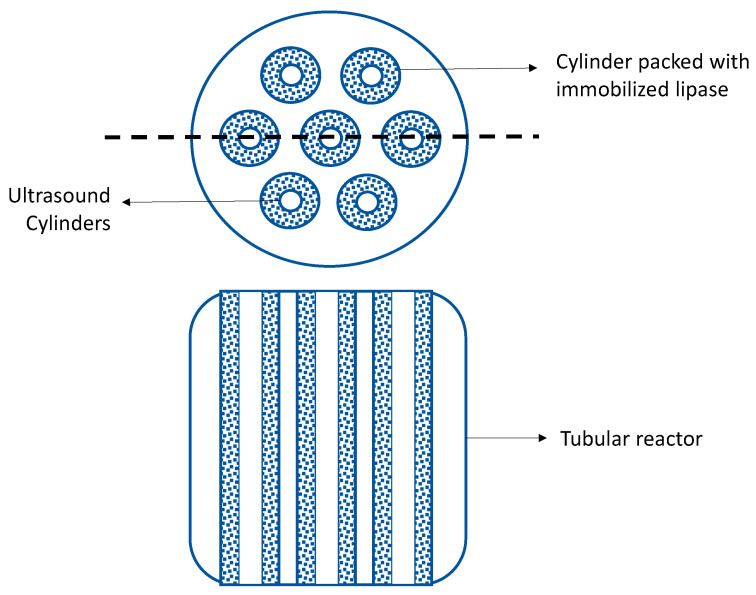
Utility model for obtaining biodiesel using used cooking oil and ultrasound as a substrate [33].

Another line of working in granted patents is the use of wasted cooking oil as substrate for the enzymatic processes. To build innovation and IP, this use is combined with process development approaches. For example, the design of a jacketed cylindrical reactor, in which several tubes are inserted, and packed inside with an immobilized lipase. An ultrasound system passes through the center of each tube (concentric) (Figure 4). This utility model allows the reaction system to be intensified, reducing reaction times and improving the economics of the process [33].

Within utility models, some innovations in reactors have been disclosed, e.g., the use of an autoclave to conduct enzymatic transesterifications combined with a heating system provided by a heat-jacket and stirring, and with a pumping system to enable a better mixing. Remarkably, the autoclave can be used in series with others, and the separation of the immobilized enzyme can be carried out with a disk centrifuge. The system can be scaled, and the advantage with conventional reactors is an increase in the reaction rate, increasing efficiency and saving energy [34].

In the field of biodiesel and biocatalysis, some innovations have also been created in the enzymatic production process. For instance, the chemoenzymatic esterification and transesterification process to obtain biodiesel has been reported. In a first stage, an enzymatic esterification is carried out with a commercial lipase to valorize the free fatty acids (FFA) as substrates, which otherwise may be lost through a classic base-catalyzed biodiesel synthesis. Subsequently, the transesterification of the esters generated in the first stage is performed with sodium hydroxide (avoiding the risk of saponification of the FFAs with the base). This methodology allows the quantity of product to be increased while maintaining low production costs, and with a potential for development on an industrial scale [35].

Finally, it is worth mentioning that innovation in enzymatic technology has also been applied for the production of motor oils, specifically in modifying vegetable oils and grease. An example is the modification of castor oil by means of an enzymatic esterification of the acidic compounds of vegetable oil, which constitutes 10% of the final formulation of motor oil. In this sense, enzymatic processes are considered as an interesting alternative for obtaining industrial additives [36].

When comparing all these patents, it is reflected that most of them focus on the engineering of the biocatalyst, either by means of recombinant DNA modifications or immobilization in order to increase its stability and reuse. Other innovative lines are the set-up of novel reactors and apparatus.

## 3. Recent Granted Patents Related to Biotransformations and Sugar Commodities

The global industrial sugar market is projected to reach USD 52.91 billion by 2022, growing at a compound annual growth rate (CAGR) of 6.01% from 2016 to 2022. Sugars are considered to be among the most important commodities and are produced and consumed worldwide. From the global demand, around 70% is consumed in domestic markets and only 30% is traded internationally [37]. Currently, sugar reduction is a global consumer trend, and governments worldwide have also implemented policies to reduce sugar consumption, with an increase in the market for alternative sweeteners [38]. These changes require new innovations, where biotransformations may be essential for the development of sustainable and efficient processes, thanks to the excellent regioselectivity that enzymes can display, and given the complexity that sugar chemistry exerts. 

**Figure 5:**
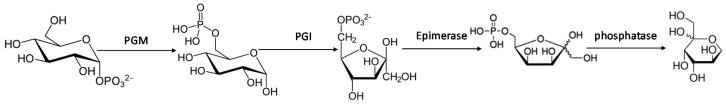
Enzymatic cascade reaction to obtain *D*-Tagatose [38].

Innovation has been reported in the enzymatic production of *D*-tagatose, with a process involving an enzymatic cascade reaction. The first step converts a saccharide to the glucose 1-phosphate (G1P), which is subsequently converted to glucose 6-phosphate (G6P) catalyzed by phosphoglucomutase (PGM). Then, G6P is converted to fructose 6-phosphate (F6P), catalyzed by phosphoglucose isomerase (PGI), and F6P is converted to tagatose 6-phosphate (T6P), catalyzed by an epimerase. Finally, T6P is converted to tagatose, catalyzed by a phosphatase (Figure 5). 

The saccharides used as substrates in this process can be selected from the group consisting of a starch or its derivative, cellulose or its derivative and sucrose. The advantage is that it can be carried out in a bioreactor or in serial bioreactors, which confers promising economic perspectives. Another advantage is the use of low-cost starting materials, and the reduction in costs associated with the feedstock and product separation. Starch, cellulose, sucrose and their derivatives are less expensive feedstocks than, for example, lactose. When tagatose is produced from lactose, glucose, galactose and tagatose are separated via chromatography, and the production costs are higher [39,40]. Another multienzyme system to produce *D*-tagatose adds α-amylase and cellulase enzymes to the raw material before being converted to D-tagatose in the same way as the previous methodology [40].

In the area of saccharides and biotransformations, another interesting alternative is the use of cell lysates to obtain sugars (e.g., allulose, glucose, fructose, sorbitol, ribulose, ribose and/or arabinose). Herein, the innovation is to use cell lysates from different cultures of genetically modified crops to obtain thermostable enzymes. The advantage of this system is that the enzymatic reactions are irreversible, which allows the achievement of high yields. Conversely, typical conventional biotransformation methods are thermodynamically controlled reversible processes, which prevents high yields from being reached. For example, the isomerization of glucose to fructose has an approximate yield of 45%, and the epimerization of fructose to allulose has an approximate yield of 20%, a situation that requires expensive separation steps [41].

**Figure 6:**
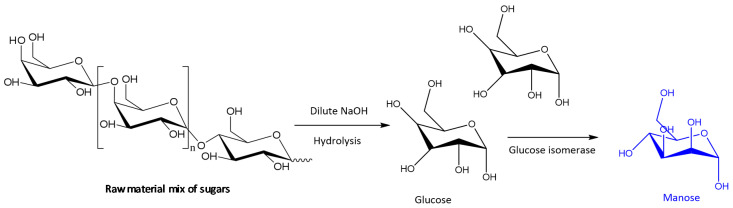
Enzymatic process to obtain mannose from raw material [42].

Obtaining mannose by means of a chemoenzymatic system has also been patented recently. The process consists of a first stage in an alkaline hydrolysis at low concentrations to obtain glucose and fructose from the raw material. Then, in the enzymatic stage, the enzymes glucose isomerase and mannose isomerase catalyze the reactions to obtain mannose. Purification of the final product is carried out by chromatography (Figure 6) [42].

The enzymatic production of D-Psicose is also patented and consists of the biotransformation of fructose to D-Psicose, using the enzyme D-Psicose 3-epimerase as biocatalyst. Then, in downstream processes, residual fructose is transformed into gluconic acid catalyzed by the enzymes glucose isomerase and glucose oxidase. In this way, this gluconic acid is adsorbed on an ion exchange column, obtaining D-Psicose at high concentrations through a simple method with industrial application, with straightforward downstream processing [43].

**Figure 7:**
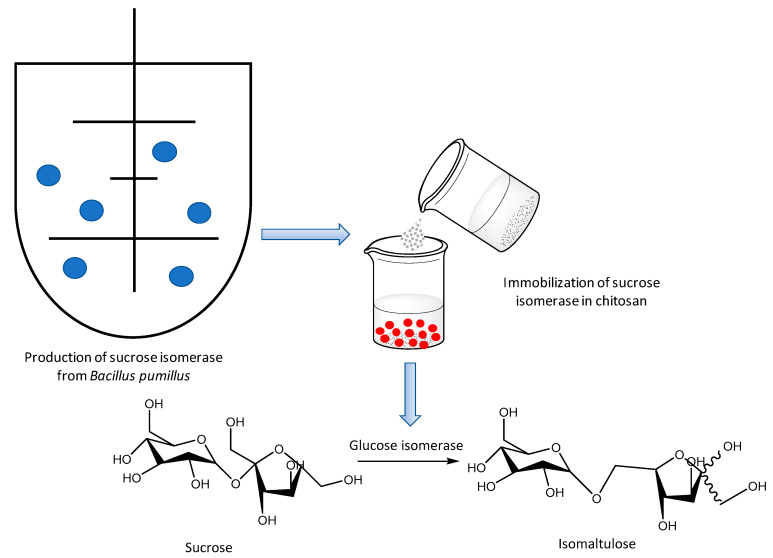
Enzymatic process to obtain isomaltulose from sucrose [44].

Apart from the emphasis on reactions (see above), enzymatic immobilization methodologies for the production of sugars have also been protected by granted patents. This is the case of the immobilization of recombinant sucrose isomerase, whose gene was expressed in the microorganism *Bacillus pumilus*. The immobilization of sucrose isomerase was carried out on chitosan microspheres, which enabled an enhancement of the thermal and pH stability of the biocatalyst to achieve an isomaltulose yield of >87%, resulting in a process with industrial application (see Figure 7) [44].

In the biotransformation of sugars related to granted patents, ionic liquids (IL) have also been used as a reaction medium to produce fructose catalyzed by the enzyme glucose isomerase. The IL, 3-methylimidazole proline can be recycled in the process. The fructose yields achieved by this methodology range from 35 to 37% [45].

In the production of glucose from cellulose, innovative strategies have been disclosed, e.g., applying a chemoenzymatic process which consists of an acid or alkaline pretreatment at low concentrations, then using cellulases (used to hydrolyze the *β*-1,4-glucan or *β-*D-glucosidic to shorter oligosaccharides). The yields obtained range from 50 to 95% and after the downstream process, 98% cellulose purity is reached [46].

## 4. Recent Granted Patents Related to Bioprocesses for Ester Production

**Figure 8:**
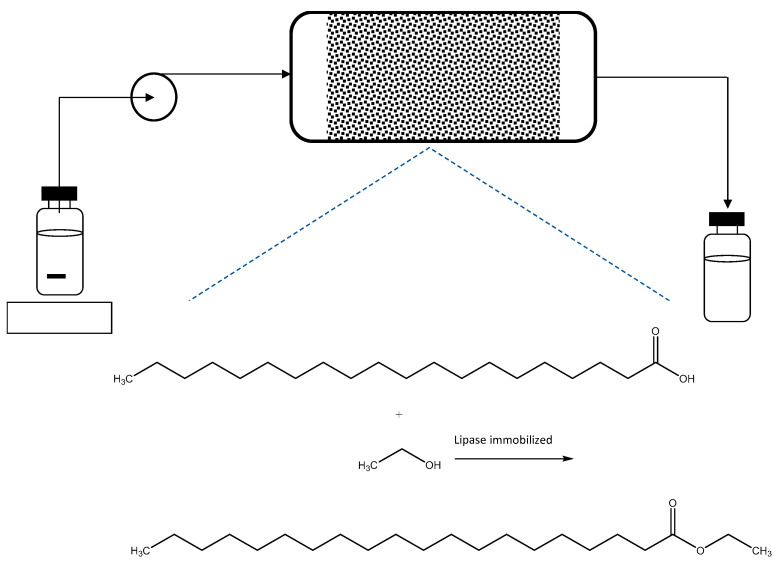
Production of docosahexanoic (DHA) and eicosapentaenoic acid (EPA) in a continuous packed-bed enzymatic bioreactor [48].

Esters are important bulk additives in both the cosmetic and food industries. Over the last five years, different inventions related to biotransformations and esters have been disclosed in the form of granted patents [47]. For example, the development of a continuous process in a packed-bed bioreactor for the production of docosahexanoic (DHA) and eicosapentaenoic acid (EPA) esters catalyzed by an immobilized lipase (Figure 8) has been implemented. The raw materials used in this process are ethanol and fats and oils rich in DHA and EPA. However, the batch reactor presents the disadvantages of reaching low conversions and rates. In order to overcome these hurdles, a continuous packed-bed process was developed which allowed the reaction rate to be increased, reaching a conversion greater than 90% at 60 °C with a residence time ranging between 0.1 and 60 min [48].

The production of monoglycerides catalyzed by an enzymatic alcoholysis has also been protected a Korean granted patent. The monoglyceride produced is 2-palmitoyl glycerol, and the raw materials can be different animal and vegetable fats. The process consists of a pre-treatment of the raw material (fat) with the solvent (ethyl alcohol) up to temperatures of 70 °C. Then, the temperature is lowered to 40 °C to enable the transesterification catalyzed by immobilized *Candida antarctica* lipase B (CALB). The innovation of this process is the pre-treatment at high temperatures, which allows higher yields than conventional systems to be achieved [49].

**Figure 9:**
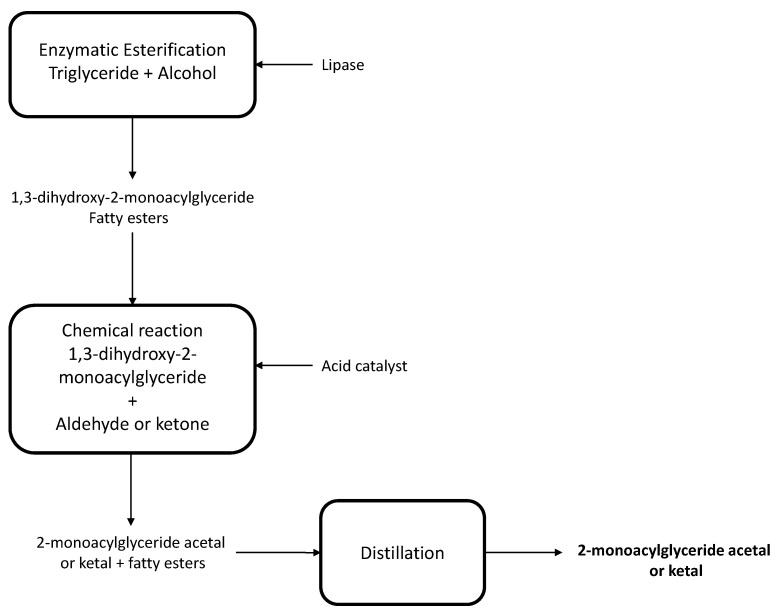
Flow chart of the combined chemoenzymatic process to render 2-monoacylglyceride [50].

In general, the production of 2-monoglycerides poses challenges, since secondary alcohols, present in glycerol, are less reactive than primary alcohols. Additionally, the 2-monoglycerides are thermodynamically susceptible to suffer acyl migrations, forming 1,3 monoglycerides. This challenge has been recently addressed in a granted patent (Figure 9) [50]. The method comprises three steps. An unsaturated triglyceride is first reacted with at least two molar equivalents of water, a C1–C8 alcohol in the presence of a lipase at a temperature within the range of 20 to 80 °C to produce a mixture of 1,3-dihydroxy-2-monoacylglyceride and fatty esters or acids. The 1,3-dihydroxy-2-monoacylglyceride is then reacted with at least one molar equivalent of an aldehyde or ketone, optionally in the presence of an acid catalyst, to produce a mixture comprising a 2-monoacylglyceride acetal or ketal. Next, the fatty esters or acids are removed from the mixture as an overhead product by distillation or wiped-film evaporation to isolate a purified 2-monoacylglyceride acetal or ketal. Preferably, the 2-monoacylglyceride acetal or ketal is further purified by distillation to separate it from less volatile impurities. Food grade aldehydes or ketones such as acetaldehyde, hexanal, octanal, benzaldehyde, cinnamaldehyde, vanillin, ethyl vinyl ketone, 2-furyl methyl ketone, methyl 2-pyrrolyl ketone, watermelon ketone and raspberry ketone are preferred. Organic acid catalysts such as acetic acid or *p-*toluen-sulfonic acid are preferred. An important aspect of this technology is that it makes use of distillation as a separation process, which allows the process to be scaled up, in contrast to more challenging chromatographic separations [50].

An interesting innovation that takes advantage of the by-products of the biodiesel process consists of the reaction of the formed by-products (glycerol and fatty acids) that are enzymatically esterified with diethyl carbonate, generating a polybasic unsaturated fatty acid ester. This technique constitutes a clean process, which generates a product of good quality and high performance, while recycling by-products and improving overall economics of the process [51].

Due to their nutritional characteristics, structured triglycerides are important as food additives and supplements for human health. An innovation for the production of triglycerides has been patented, consisting of the reaction of a 2-monoglyceride as an acyl acceptor with an acyl donor as a vinyl ester of acid grade, catalyzed by a lipase. The reaction results in an asymmetric triglyceride, and compared with conventional methods, it has many advantages, such as lower reaction temperature, higher reaction efficiency and lower costs for raw material, which result in an overall industrially sound and simpler approach. Furthermore, another advantage of this invention is that it can be worked solvent-free or with more benign solvents, such as ethyl acetate or acetone [52].

According to the BCC research’s recent report, the global market for flavors and fragrances was valued at USD 26 billion in 2015 and is expected to reach USD 37 billion by 2021 [53]. Cosmetics takes an even larger share of the global market, valued at over USD 500 billion in 2017, and its market is expected to achieve over USD 805 billion by 2023 [54]. The increasing preference for using natural and sustainable products pushes large fragrance and ingredient firms to find alternative sources for the production of natural ingredient [55]. Given the growing commercial interest in the sustainable production of these compounds, there are innovations, such as the enzymatic obtaining of 3-hydroxy-2-butanone fatty acid ester. The reaction consists of the esterification of 3-hydroxy-2-butanone reacting with the aliphatic acid, such as caproic acid, enanthic acid, octanoic acid, n-nonanoic acid, capric acid or laurate catalyzed by immobilized *Candida antarctica* lipase B. The reaction is carried out at 50 °C with hexane as the solvent. For example, using laureate as a substrate, ca. 17 g L^−1^ of 3-hydroxy-2-butanone laurate is obtained [56]. Likewise, α-linolenic acid is an essential nutrient for human health and nutrition. Due to its important properties, innovations such as the enzymatic synthesis of α-linolenic acid monoglyceride have been found. The source of α-linolenic acid is made up of vegetable oils rich in α-linolenic acid which react with alcohol (ethyl alcohol, propyl alcohol and butanol) in low concentration (~10%) catalyzed by Sn-1,3-specific lipases, such as immobilized lipase B from *Candida antarctica* (Nov-435). The reaction mixture contains high-purity α-linolenic acid monoglyceride, after downstream filtration and molecular distillation processes. The characteristics of this new technology make the most of vegetable oil, and compared to conventional processes, the operating time and the production costs are reduced [57].

When comparing the new technologies in the production of esters, lipases, and especially lipase B from *Candida antarctica*, are the most widely used. It should also be noted that the application of these esters is in the cosmetic and nutraceutical industry.

## 5. Biotransformations with Furans to Obtain Bulk Compounds

Furans are chemicals obtained from sugars derived from lignocellulose (xylose and glucose), which form a versatile synthetic platform that can be valorized from biorefineries. Their inherent instability creates opportunities for their upgrading by using enzymes and whole cells [58], since severe reaction conditions may degrade the chemicals, leading to decreased yields, waste formation and cumbersome downstream processing units.

Several patents related to White Biotechnology have been granted in this area in recent years. Starting from glucose (from lignocellulose), the use of glucose isomerases to generate mixtures of glucose–fructose has been reported, in combination with biphasic media to produce HMF out of the fructose, as the dehydration of fructose is more straightforward than the dehydration of the original glucose [59]. In another patent, combined methods to produce hydroxymethylfurfural (HMF) and gluconic acid from fructose–glucose mixtures have been protected. While an enzymatic approach is intended for the production of gluconic acid (using glucose oxidase–catalase systems), the HMF is produced via acidic catalysis upon dehydration of fructose [60]. 

In a different approach, the use of whole cells for the valorization of HMF has also been recently granted in patents. To that end, microorganisms able to accept high loadings of furans are needed. Herein, a *Comamonas testosteroni* strain is able to accept up to 160 mM of HMF, which is useful for the further partial oxidation to render 5-hydroxy-methylfuroic acid [61]. Following these premises, the ultimate product derived from HMF is 2,5-furandicarboxylic acid (FDCA), which may become a replacement in future plastics. Herein, the synthesis of FDCA has been protected by using different HMF dehydrogenases to perform the oxidative steps from HMF to FDCA, such as the intermediates. The novelty of that patent relies on the sequence of the enzymes, and different homologies are provided [62]. Another patent combines the use of HMF oxidoreductases with HMF production and membrane technology [63]. Furthermore, a multistep enzymatic process to produce FDCA from HMF has been reported, involving xanthine oxidoreductases, galactose oxidases, aldehyde dehydrogenases and ketoreductases, to generate furan intermediates until FDCA [64].

## 6. Conclusions

Biocatalysis is attracting an increasing interest in areas related to bulk chemicals, segments that traditionally have not been central for enzyme catalysis. The drivers for using enzymes in these fields are related to energy savings, the generation of less by-products and manageable wastes, as well as the possibility of entering into new markets by using natural catalysts. Herein, an overview of selected applications in topics related to biofuels and biorefineries, ester synthesis and furan chemistry has been presented. Successfully granted applications focus on novel enzymes, immobilization methods, as well as novel devices and reactors. 

It may be expected that more innovative solutions will be reported by using enzymes in the coming years.
